# Lysozyme amyloidosis – a case report and review of the literature 

**DOI:** 10.5414/CNCS108538

**Published:** 2015-12-28

**Authors:** Christopher Pleyer, Jan Flesche, Fahad Saeed

**Affiliations:** Department of Medicine, Cleveland Clinic Foundation, OH, USA

**Keywords:** lysozyme amyloidosis, pericardial involvement

## Abstract

Lysozyme amyloidosis is an exceedingly rare hereditary autosomal dominant amyloidosis, which is characterized by the precipitation of lysozyme protein within the body, leading to multi-organ dysfunction. Herein, we present the case of a U.S. family affected by lysozyme amyloidosis. In particular, we report pericardial disease involvement leading to recurrent pericardial effusion, which to our knowledge has not been described yet. To our knowledge, we have also for the first time identified the amyloidogenic component of lysozyme amyloidosis via laser microdissection and mass spectrometry from a bone marrow biopsy. The diagnosis of this disease remains challenging as it can be easily mistaken for primary amyloidosis, which also presents with similar symptoms. Immunohistochemical staining of tissue for specific amyloidogenic proteins allows for an accurate diagnosis and should be performed in all amyloidosis patients in order to spare patients from potentially futile or harmful therapy. The widespread systemic involvement of lysozyme amyloidosis currently provides limited options for treatment, although kidney and/or liver transplantation appear to be promising palliative treatments. Practicing clinicians and researchers need to collect more information about this rare entity to further characterize the behavior of this disease and develop new potential treatment strategies.

## Introduction 

Amyloidosis is a disorder in which a normally soluble protein is misfolded and deposited as insoluble product in the extracellular space. The resulting accumulation of abnormal protein eventually leads to disruption of organ structure and function, ultimately ending in multi-organ failure. Lysozyme Amyloidosis is a rare, hereditary, autosomal dominant systemic disease characterized by diffuse precipitation of lysozyme, a ubiquitous bacteriolytic enzyme, synthesized by hepatocytes and macrophages [1, 2]. Particularly affected organs include salivary glands, gastrointestinal tract, and vasculature – leading to sicca syndrome, diarrhea, and spontaneous bleeding respectively. The diagnosis of lysozyme amyloidosis remains challenging given significant overlap of symptoms with primary AL-amyloidosis [3]. Due to lack of curative treatment options, medical management is currently focused on symptom management. 

## Case report 

**Figure 1.**
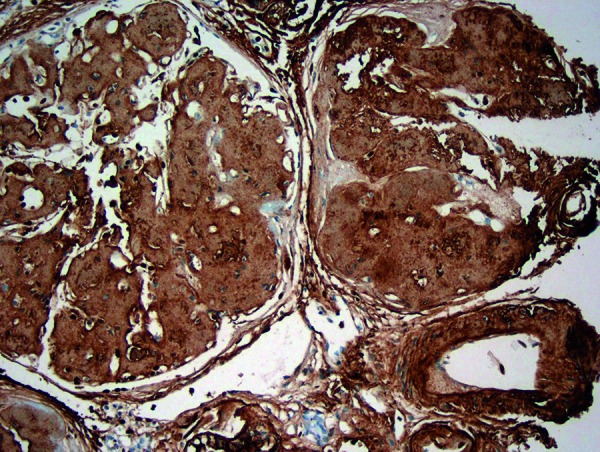
Extensive lysozyme deposition within the kidney interstitium, perivascular region and the glomeruli (brown color indicates areas of lysozyme tissue infiltration).

A 35-year-old Caucasian male with no significant previous medical history and a positive family history for lysozyme amyloidosis was admitted for 1 week history of progressive abdominal pain and acute kidney injury. Physical exam was pertinent for periorbital ecchymosis (“raccoon eyes”) and mildly tender abdomen. Initial laboratory values were concerning for acute kidney injury (creatinine 7.3 mg/dL, glomerular filtration rate < 15 mL/min). Complete blood count demonstrated mild normocytic anemia but was otherwise unremarkable (hemoglobin 10.4 g/dL, platelets 318,000, white blood cell count 74,000). He had nephrotic range proteinuria (5.6 g/24 h). Serum and urine protein electrophoresis were normal. Serum free light chain assay demonstrated mildly elevated κ/λ-free light chains (κ 74, λ 73, Ratio 1.01). Renal biopsy with semi-quantitative immunohistochemical staining was negative for IgG, IgA, IgM, κ, λ, C3c, and C1q but was strongly positive for lysozyme in the interstitium and peri-vascularly, and weakly positive in the glomerular space, confirming the diagnosis of lysozyme amyloidosis (Figure 1). A bone marrow biopsy was also performed which was positive for lysozyme involvement predominantly in the perivascular regions. Given the positive family history, we decided to perform further investigational analysis and conducted liquid chromatography mass spectrometry analysis of laser microdissected bone marrow tissue, which confirmed lysozyme C as the specific amyloidogenic protein. 

**Figure 2.**
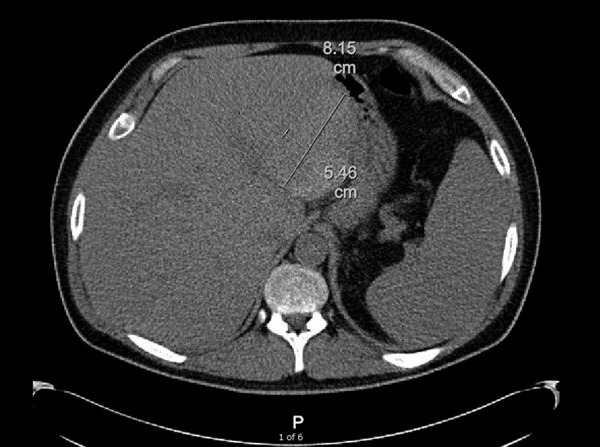
CT-Abdomen demonstrating spontaneous intra-hepatic hemorrhage, measuring 8.15 cm × 5.46 cm.

He was subsequently started on intermittent hemodialysis for progressive renal failure. Throughout his hospital course, he was complaining of worsening abdominal pain and received a CT-abdomen, which demonstrated a subcapsular renal hematoma (5.9 × 2.7 × 4.8 cm), which was likely a complication from his kidney biopsy. Interestingly, he had also developed two spontaneous liver hematomas (8.15 × 7.7 × 1.9 cm and 8.2 × 5.5 × 7.2 cm), spontaneous left adrenal hemorrhage (5.2 × 3.4 × 2.5 cm), as well as large mesenteric lymph nodes consistent with spontaneous intra-nodal hemorrhage (7.2 × 4.6 cm) (Figure 2). After extensive hematologic evaluation, including normal thromboelastography and prothrombin mixing studies, his bleeding diathesis was ascribed to disruption of vessel wall integrity from amyloid deposition. He continued to receive supportive care with intermittent hemodialysis and blood transfusions and was eventually discharged in stable condition. A routine pre-transplant echocardiogram demonstrated a large circumferential pericardial effusion with preserved ejection fraction and no hemodynamic compromise and is being managed conservatively. He currently remains on dialysis and is undergoing evaluation for kidney and liver transplantation. 

### Family history 

The patient’s mother and brother have previously been diagnosed with lysozyme amyloidosis. He has one brother and one daughter who are thought to be healthy. His brother was diagnosed with lysozyme amyloidosis at the age of 30 years after presenting with a long-standing history of sicca syndrome and worsening renal function. He had biopsy proven involvement of the kidney, liver, and heart. He eventually required hemodialysis for kidney failure. His liver and cardiac function were normal, however he developed recurrent non-hemorrhagic pericardial effusions requiring intermittent pericardiocentesis. He ultimately expired 8 years after the initial diagnosis at the age of 38 years due to a catastrophic intra-abdominal bleeding episode. 

Interestingly, the patient’s mother had a relatively indolent course. She was initially diagnosed with lysozyme amyloidosis with biopsy proven gastrointestinal and bone marrow involvement at the age of 62 years after presenting with sicca syndrome, mild gastrointestinal bleedings, as well as rapidly progressive renal failure necessitating a kidney transplant in the same year of the diagnosis. Her post-transplant course was complicated by a spontaneous large liver hematoma that spontaneously regressed. Her renal allograft has stable function more than 10 years post-transplant. 

## Discussion 

In this report we describe a family with hereditary lysozyme amyloidosis, mainly characterized by renal, gastrointestinal, and cardiac involvement. Lysozyme amyloidosis is generally thought to be a slowly progressing disease with a median survival rate of 17.9 years after diagnosis [4, 5]. However, there have been reports of wide-spread phenotypic heterogeneity between subjects [5, 6, 7]. Interestingly, subjects involved in this case report have demonstrated features of both slow progression in the mother, as well as rapid progression after the initial diagnosis in both sons. The mechanism of an accelerated course of disease remains relatively unclear. 

The current gold standard for diagnosing any type of amyloidosis is immunohistochemical staining of tissue for specific amyloidogenic proteins. Potential further diagnostic and prognostic testing which are currently mainly performed for research purposes include testing for specific DNA mutations, scintigraphic imaging for serum amyloid P (SAP), and laser-microdissection with mass spectrometry. In this study, we confirmed lysozyme C as the specific amyloidogenic protein via laser microdissection with mass spectrometry of the bone marrow sample. To our knowledge, this is the first report of amyloidogenic protein being identified in the bone marrow. In regards to mutational analysis, four different DNA mutations have been attributed to lysozyme amyloidosis so far, however there are currently no known prognostic or therapeutic implications based on specific DNA mutations [5]. Scintigraphic imaging for SAP, rarely performed in the United States, has been used as a non-invasive test to identify the site(s) and extent of organ involvement in Europe. Scintigraphic imaging could be of value in providing prognosis and guiding decisions for potential organ transplantation [8]. Importantly, lysozyme amyloidosis is often hard to distinguish from primary AL-amyloidosis. One study found that 34 of 350 subjects (~ 10%) previously diagnosed with primary AL-amyloidosis, in fact, had suffered from a hereditary form of amyloidosis one of which was lysozyme amyloidosis [3]. In order to avoid misdiagnosis and potential futile and/or harmful treatments, it is imperative to identify the involved protein in all amyloidosis cases via tissue biopsy and immunohistochemical staining prior to establishing a definite diagnosis. 

The main mechanism driving complications in lysozyme amyloidosis is disruption of organ structure and function by amyloid deposition. Common symptoms are sicca, diarrhea and worsening kidney function caused by infiltration of salivary glands, gastrointestinal tract, and kidneys respectively. Amyloid deposition in the kidney can occur anywhere. The strongest deleterious effect on kidney function is in the tubulointerstitium where it causes atrophy and fibrosis [9]. There are reports that amyloidogenic proteins have a direct toxic effect independent of amyloid deposition. This, however, has only been shown in in-vitro studies for AL-amyloidosis [10] and it is uncertain if this is also the case for lysozyme amyloidosis. 

A number of cases in the literature also describe a predilection of lysozyme to target liver and vasculature [6, 7]. This can be explained by the fact that lysozyme is produced by hepatocytes and macrophages, thereby increasing the disease burden in these organs. Interestingly, despite diffuse organ involvement, liver function appears to be minimally compromised in lysozyme amyloidosis. In contrast, vasculature is vulnerable to disruption by lysozyme infiltration, thereby, increasing the risk for spontaneous bleeding, as seen in our case. Acquired hemostatic defects are rare in lysozyme amyloidosis [11] and the main process driving the propensity to bleeding is via lysozyme mediated breakdown of vessel wall integrity [4]. 

In addition to typical target organ damage, our report highlights a rare complication of this disease i.e., cardiac involvement complicated by pericardial effusions. Given the history of biopsy proven cardiac involvement in his sibling, we presume that our patient’s pericardial effusion is also due to amyloidosis, though he did not undergo a pericardial biopsy. Uremic pericardial effusion from renal failure is certainly another likely differential diagnosis and cannot be excluded definitively. Uremia related pericardial effusion tends to resolve after dialysis initiation, which did not occur in our patient leading amyloidosis as the likely etiology. The mechanism through which pericardial effusions develop in amyloidosis patients is currently uncertain and warrants further research. 

To date, there is no curative therapy for lysozyme amyloidosis and treatment is largely supportive. Kidney transplantation has been available to a subset of patients suffering from various types of amyloidosis. A study of 104 amyloidosis patients, including 3 subjects with lysozyme amyloidosis, reported no transplant failure after a median of 2.9 years of follow-up [12]. In addition, no relationship between extra-renal organ involvement or total amyloid burden and overall transplant survival was identified. Liver transplantation may also be beneficial as a palliative and life-prolonging measure. Two studies have reported successful liver transplantation in 5 patients, showing no evidence of hepatic graft failure 1.7 – 11 years post-transplant [5, 7]. 

In conclusion, lysozyme amyloidosis is a disease that continues to pose challenges to all the stakeholders including patients, physicians, and researchers. There is a need to collect more knowledge about this rare entity to further characterize the natural course and pathophysiology of this disease to develop new potential treatment strategies. 

## Conflict of interest 

The authors have no conflict of interests.
